# Roles of the MPFC and insula in impression management under social observation

**DOI:** 10.1093/scan/nsab008

**Published:** 2021-01-15

**Authors:** Leehyun Yoon, Kwangwook Kim, Daehyun Jung, Hackjin Kim

**Affiliations:** Department of Human Ecology, University of California, Davis, CA 95616, USA; Laboratory of Social and Decision Neuroscience, Korea University, Seoul 02841, Republic of Korea; Department of Psychology, Korea University, Seoul 02841, Republic of Korea; Laboratory of Social and Decision Neuroscience, Korea University, Seoul 02841, Republic of Korea; Department of Psychology, Korea University, Seoul 02841, Republic of Korea; Laboratory of Social and Decision Neuroscience, Korea University, Seoul 02841, Republic of Korea; Department of Brain and Cognitive Engineering, Korea University, Seoul 02841, Republic of Korea; Laboratory of Social and Decision Neuroscience, Korea University, Seoul 02841, Republic of Korea; Department of Psychology, Korea University, Seoul 02841, Republic of Korea

**Keywords:** fMRI, medial prefrontal cortex, self-enhancement, self-referential processing, insula

## Abstract

People often engage in impression management by presenting themselves and others as socially desirable. However, specific behavioral manifestations and underlying neural mechanisms of impression management remain unknown. In this study, we investigated the neural mechanism of impression management during self- and friend-evaluation. Only participants assigned to the observation (OBS) group, not the control (CON) group, were informed that their responses would be monitored. They answered how well positive and negative trait adjectives described themselves or their friends. The behavioral results showed that the OBS group was more likely to reject negative traits for self-evaluation and to accept positive traits for friend-evaluation. An independent study revealed that demoting negative traits for oneself and promoting positive traits for a friend helps manage one’s impression. In parallel with the behavioral results, in the OBS *vs* the CON group, the rostromedial prefrontal cortex (rmPFC) and anterior insula (AI) activity showed a greater increase as the negativity of negatively valenced adjectives increased during self-evaluation and also showed a greater increase as the positivity of positively valenced adjectives increased during friend-evaluation. The present study suggests that rmPFC and AI are critically involved in impression management, promoting socially desirable target evaluations under social observation.

## Introduction

Impression management, which is ‘the process by which individuals attempt to control the impressions others form of them’ (Leary and Kowalski, 1990), plays a critical role in many interpersonal behaviors. People strategically use self- and other-evaluation to promote favorable self-images (Sedikides *et al.*, 2007; Bolino *et al.*, 2008) by softening positive self-description (Schütz, 1997; Exline and Lobel, 1999; Sedikides *et al.*, 2002), reducing internal attribution of success and increasing their acknowledgment of others’ contributions to achievements (Miller and Schlenker, 1985; Baumeister and Ilko, 1995). Supporting the function of these behaviors in transmitting favorable impressions, evidence has shown that those who avoid extreme negative or positive self-descriptions (Schlenker and Leary, 1982; Powers and Zuroff, 1988; Robinson *et al.*, 1995; Wosinska *et al.*, 1996), enhance others (Gordon, 1996) and behave modestly (Wang and Highhouse, 2016) are liked by others, while individuals who are self-deprecating (Lai *et al.*, 2010; Wang and Highhouse, 2016) and excessively self-enhancing (Colvin *et al.*, 1995; Paulhus, 1998) are viewed as unattractive by others. Despite the prevalence and significance of impression management in maintaining social relationships, its neural mechanisms remain poorly understood. The present study aimed to identify the neurocircuitry of impression management that underlies increased socially desirable self- and other-evaluation.

While few studies have investigated the neural mechanisms of impression management, several studies have reported the neural mechanisms associated with key factors closely related to it, including social observation (Leary and Kowalski, 1990), socially desirable behavior (Leary *et al.*, 2011) and self-conscious emotions (Doherty and Schlenker, 1991; Leary, 2004). Notably, the rostromedial prefrontal cortex (rmPFC) has been consistently associated with these factors.

First, social observation, which often facilitates impression management, elicits rmPFC activity. For example, rmPFC activity increases when judging the self and social appropriateness under social observation (Izuma *et al.*, 2010), when receiving feedback on success or failure in public (Müller-Pinzler *et al.*, 2015) and when playing an economic game under social observation (Van Hoorn *et al.*, 2016). In addition, trait social anxiety or dispositional sensitivity to anticipated social evaluation was positively associated with rmPFC activity during social observation (Müller-Pinzler *et al.*, 2015), and increased rmPFC activity during social observation was accompanied by self-reported self-conscious emotions (Somerville *et al.*, 2013). rmPFC activity has also been shown to encode the value of prosocial decisions under social observation (Jung *et al.*, 2018).

Second, rmPFC activity has been associated with norm-compliant or socially desirable behaviors, a typical way of expressing impression management, especially in the absence of specific instructions regarding an audience’s traits or values (Leary *et al.*, 2011). Specifically, an increase in rmPFC activity was associated with more generous donations (Hare *et al.*, 2010; Tusche *et al.*, 2016), more frequent daily helping behavior (Rameson *et al.*, 2012) and an increase in purchasing ethical products (Jung *et al.*, 2018). rmPFC activity has also been linked to compliant behavior due to social influence, such as an increase in sunscreen use (Falk *et al.*, 2010) and reduced smoking behavior (Cooper *et al.*, 2015; Pegors *et al.*, 2017) after receiving persuasive messages.

Third, rmPFC activity has been associated with self-conscious emotions, which are often elicited by concerns about how one is perceived by others (Leary, 2004) and whether one’s behavior is aligned with social standards (Tracy and Robins, 2004; Tangney *et al.*, 2007). Previous studies have linked rmPFC activity to various types of self-conscious emotions, such as embarrassment (Takahashi *et al.*, 2004; Bas-Hoogendam *et al.*, 2017), shame (Michl *et al.*, 2012), guilt (Zahn *et al.*, 2008; Basile *et al.*, 2011; Wagner *et al.*, 2011; Gilead *et al.*, 2016) and pride (Zahn *et al.*, 2008). Brain lesion studies and structural neuroimaging data also support the role of the rmPFC in self-conscious emotions. Specifically, patients with brain lesions in the rmPFC failed to exhibit self-conscious emotions (Sturm *et al.*, 2006, 2008; Krajbich *et al.*, 2009) and engage in socially inappropriate self-disclosure (Beer *et al.*, 2006). Furthermore, reduced rmPFC volume was associated with reduced embarrassment, as measured by physiological responses and facial expressions while watching oneself singing in front of others (Sturm *et al.*, 2012).

In addition to the rmPFC, the anterior insula (AI) can also be related to impression management. Increased AI–rmPFC functional coupling predicted prosocial behavior under social observation (Jung *et al.*, 2018), and people with social anxiety or social phobia exhibited more activity in these two regions when receiving social feedback (Heitmann *et al.*, 2014) and when reading stories of embarrassing social transgressions (Blair *et al.*, 2010). Moreover, patients with behavioral variant of frontotemporal dementia, which is characterized by neurodegeneration in the AI and rmPFC (Seeley, 2010; Ibañez and Manes, 2012), show a lack of embarrassment following inappropriate social behavior (Snowden *et al.*, 2001; Sturm *et al.*, 2006, 2008; Moll *et al.*, 2011).

Based on these findings, we predicted that the rmPFC and AI would be implicated in impression management under social observation. While the functional division of the mPFC has not been clearly defined, recent studies have suggested that the rmPFC has a unique function in self-monitoring based on social standards (Dixon *et al.*, 2017) and is functionally distinguished from the other adjacent regions, such as the dorsomedial prefrontal cortex and ventromedial prefrontal cortex, which are implicated in mentalization and affective processing or representing integrated situational knowledge, respectively (De La Vega *et al.*, 2016; Lieberman *et al.*, 2019). We defined the rmPFC as an mPFC subregion vertically located between *z* = −10 and *z* = 20 in the Montreal Neurological Institute (MNI) coordinates (Dixon *et al.*, 2017) and caudally extended to the genu of the corpus callosum, thus covering clusters from many studies involving self-conscious emotions (Somerville *et al.*, 2013; Bas-Hoogendam *et al.*, 2017) and social observation (Izuma *et al.*, 2010).

In our experimental design, participants were randomly assigned to an observation (OBS) group or a control (CON) group and performed a trait-evaluation task in which they rated how well each trait adjective described themselves or their friends. We predicted that the trait evaluation of self and others in the OBS group would be biased toward the goal of impression management compared to that in the CON group. At the neural level, we expected that rmPFC and AI activity would be consistent with the behavioral evidence for impression management. To verify that enhanced motivation for impression management underlies the observation effect in self- and friend-evaluation, we recruited an independent sample that rated the impression of a hypothetical person who described themselves or their friends with the adjectives used for the functional magnetic resonance imaging (fMRI) task. Then, we examined whether the impression ratings varied as a function of the valence level of adjectives, consistent with the observation effect.

## Materials and methods

### Participants

Forty-five participants (mean age = 23.7, 21 males) were recruited; among them, 23 were assigned to the CON group (mean age = 23.1, 12 males) and 22 to the OBS group (mean age = 24.3, 9 males). Two participants in the OBS group were excluded from the data analyses due to excessive head movement (>3 mm), leaving 20 participants in the OBS group (mean age = 24.2, 8 males). Sample size determination is provided in Supplementary Text 1. All participants were right-handed and screened for a history of psychiatric or neurological disease and were eligible for MRI testing. Participants provided informed consent and were compensated with KRW 30,000 for their participation. The experimental procedures were approved by the institutional review board of Korea University.

### Stimuli

For the trait-evaluation task, 150 adjectives were selected from a list of personality trait adjectives used in a previous study (Anderson, 1968), which had been translated into Korean (Sul *et al.*, 2012). Valence ratings of adjectives were obtained from a previous study (Sul *et al.*, 2012), where the participants (*N *= 80) were asked to rate the social desirability of a person with each trait using a 7-point Likert scale (1 = highly undesirable to 7 = highly desirable). Among the 150 adjectives, 60 were negative (mean rating ≤3) and 60 were positive (mean rating ≥5). We analyzed the participants’ decisions regarding positive and negative adjectives separately in order to distinguish between target-promoting and target-demoting evaluations. The remaining 30 adjectives had a moderate valence level (3< mean rating <5) and served as filler trials. Descriptive statistics of valence ratings for negative, positive and filler conditions and example adjectives are presented in Supplementary table 1.

### Impression ratings from independent samples

By recruiting two independent samples for self-evaluation (*N *= 15, mean age = 24.1, 7 males) and friend-evaluation (*N *= 16, mean age = 24.4, 8 males), we measured the impression of a hypothetical person who evaluated themselves and a friend with 150 trait adjectives. Participants were instructed to rate (1 = very negative to 7 = very positive) their impression of a person who described themselves or their friends with a particular adjective (e.g. ‘I am competent’ and ‘My friend is kind’). We ensured that impression ratings of self-evaluation and friend-evaluation were obtained from two separate groups of participants to avoid potential rating contamination. While our primary purpose of collecting impression ratings was to confirm whether the valence level of self-negative (SN), self-positive (SP), friend-negative (FN) and friend-positive (FP) conditions have a role in modulating the impression, we additionally used impression ratings to provide data about the effect of the impression value of adjectives on the target evaluation of our fMRI sample (see Supplementary Text 2 for the analysis and the result).

### Experimental procedures

Participants received detailed experimental instructions and were randomly assigned to the OBS or CON groups. OBS participants were told that due to an unexpected technical problem, two researchers would monitor the experiment and manually record their responses during the task, whereas CON participants were not informed of any monitoring. In our task, the target friend was specified as one’s fifth closest friend. We avoided choosing a friend that was too close to the self to prevent overlapping representations between the self and others (Mashek *et al.*, 2003; Aron *et al.*, 2004). We also avoided choosing an unfamiliar person to ensure that the participants possessed sufficient person knowledge necessary for completing the task. To ensure that participants carried out the task by recalling their specific friends, they were asked to complete a questionnaire that reported the brief profile of their fifth closest friend and past interactions with the friend just before scanning (Supplementary Text 3).

### fMRI task

**Fig. 1.**
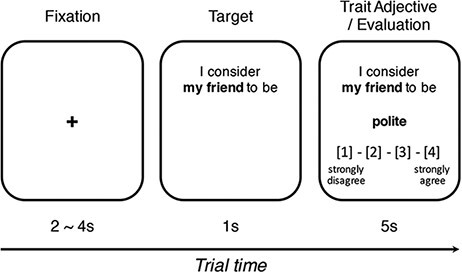
A schematic diagram of a typical trial in a trait-evaluation task during fMRI scanning. Participants were asked to evaluate how each trait adjective described themselves or their fifth closest friend. ‘My friend’ was replaced with ‘myself’ in the self-evaluation trials. The OBS group was told that two researchers would be monitoring the task, whereas the CON group was not informed of any possibility of being monitored.

The task consisted of 75 self-evaluation and 75 friend-evaluation trials. In each trial, after the fixation cross (2–4 s), a target for evaluation was presented for 1 s (i.e. ‘I consider myself to be’ or ‘I consider my friend to be’). Subsequently, a trait adjective and a 4-point rating scale were presented for 5 s (Figure 1). Participants evaluated how appropriately each trait adjective described themselves or their fifth closest friend by pressing one of four buttons corresponding to a value on the scale (1 = strongly disagree, 2 = disagree, 3 = agree and 4 = strongly agree). The self and friend conditions were presented in a pseudo-randomly mixed order. Pairings between the trait adjectives and targets were counterbalanced across participants. Cogent 2000 (www.vislab.ucl.ac.uk/cogent.php) was used to present the stimuli. The task duration was approximately 15 min. Participants were debriefed upon completion of the task. The fMRI data acquisition procedure is presented in Supplementary Text 4.

### Behavioral data analysis

For the subject-level analysis, we ran four linear regression models [equations (1)–(4)] to estimate the extent to which the valence level of positive and negative adjectives presented for self- and friend-evaluation (i.e. SN, SP, FN and FP) influenced participants’ decisions. In each model, $y$ denotes the participants’ decision about how much they agree or disagree with the applicability of the given adjective to the target. Meanwhile, $x$ denotes the valence level of the presented adjective and ${\beta _0} $ represents the intercept. The estimate of $\beta $ (i.e. $\hat \beta $) indicates the degree to which the participant’s decision was modulated by an adjective’s valence level. To make the greater *x* values represent the higher degree to which the adjectives are positive or negative, the *x* values in equations (1) and (3) were reverse-coded (e.g. 2.5 became 5.5). The *y* values of equations (1) and (3) were also reverse-coded to make the greater *y* values represent greater disagreement. Therefore, high ${\hat \beta _{SN}}$ and ${\hat \beta _{FN}}{\ }$indicate that participants were more likely to disagree as the degree of negativity of adjectives increased. High ${\hat \beta _{SP}}$ and ${\hat \beta _{FP}}{\ }$indicate that participants were more likely to agree as the degree of positivity of adjectives increased.

(1) \begin{equation*}y = {\beta _{SN}}{x_{SN}} + {\beta _0}\end{equation*}

(2) \begin{equation*}y = {\beta _{SP}}{x_{SP}} + {\beta _0}\end{equation*}

(3) \begin{equation*}y = {\beta _{FN}}{x_{FN}} + {\beta _0}\end{equation*}

(4) \begin{equation*}y = {\beta _{FP}}{x_{FP}} + {\beta _0}\end{equation*}

For the group-level analysis, four parameter estimates (${\hat \beta _{SN}}, {\hat \beta _{SP}}, {\hat \beta _{FN}}, {\hat \beta _{FP}}$) from each participant were entered into a three-way analysis of variance (ANOVA) with a between-subject factor of group (OBS or CON) and within-subject factors of target (self or friend) and valence (negative or positive). *Post hoc* analyses were conducted to confirm the observation effects under specific conditions. SPSS v.23 (IBM Corp., Armonk, NY, USA) was used for the analysis.

### Correlational analysis of independent sample data

Using data collected from the independent sample, we calculated the mean impression ratings of each trait adjective after excluding the outliers (i.e. greater than 3 SD from the mean). Four correlational analyses were performed for SN, SP, FN and FP conditions to examine whether impression ratings were modulated by the valence level of adjectives that the hypothetical speaker used for target evaluation.

### fMRI data preprocessing

Statistical Parametric Mapping 8 was used for the data analyses. The slice timing differences were corrected for calibrating the interleaved image acquisition sequence, and realignment was performed to correct for head motion with reference to the mean brain image. The resulting images were spatially normalized with respect to the MNI echo-planar imaging template, and each voxel was resampled at 2 × 2 × 2 mm resolution. The data were smoothed using a 6 mm full-width-at-half-maximum Gaussian filter.

### Definition of Regions of Interest

Based on our prediction of the role of the rmPFC and AI in impression management, we conducted the analysis by restricting the search area to the combined anatomical masks of the two regions. The rmPFC mask combined the bilateral medial orbitofrontal regions, the rectus regions, the superior medial frontal regions and the anterior cingulate cortex from the anatomical automatic labeling atlas (Tzourio-Mazoyer *et al.*, 2002), within the boundaries mentioned in the introduction (i.e. −10 < *z* < 20 and anterior to the genu of the corpus callosum). The AI mask combined the ventral and dorsal AI defined in a previous study that reported functional subdivisions of the insula (Deen *et al.*, 2010). The boundaries of the regions of interest (ROIs) in the MNI coordinates are provided in Supplementary Text 5. Anatomical masks were generated using MRIcron (http://people.cas.sc.edu/rorden/mricron/index.html) and MarsBaR (Brett *et al.*, 2002) as shown in Supplementary Figure 1.

### fMRI statistical analysis

To be in line with the behavioral analysis, we adopted an fMRI analysis that aimed to identify brain regions whose correlations with valence level are modulated by the factors of group (OBS or CON), target (self or friend) and valence (negative or positive) (see Supplementary Text 6 and Supplementary Table 2 for the alternative analyses with parametric modulators of participants’ ratings).

The general linear model (GLM) included the regressors of hemodynamic responses time-locked to four events: presentation of a (1) negative and (2) positive adjective during self-evaluation, and presentation of (3) negative and (4) positive adjectives during friend-evaluation. Each regressor was parametrically modulated by the valence level of the adjectives. The valence level of negative adjectives was reverse-coded such that a higher value indicated a more negative valence. The onset times of filler trials (i.e. moderately valenced adjectives), onset times of button press and six motion parameters were included as regressors of no interest.

In the second-level analysis, parametric modulation maps were entered into a 2 × 2 × 2 mixed factorial design, with group (OBS or CON) as the between-subject factor and target (self or friend) and valence (negative or positive) as the within-subject factors. To examine the three-way interaction effect parallel to the behavioral results, we planned the following contrasts: Self [OBS (Neg − Pos) − CON (Neg − Pos)] − Friend [OBS (Neg − Pos) − CON (Neg − Pos)].

To correct for multiple comparisons, we ran a small-volume correction (SVC) with the ROI and whole-brain gray matter. To determine the desired cluster size for surviving multiple comparison correction at α < 0.05 and an uncorrected *P*-value of 0.001, we used 3dClustSim of Analysis of Functional NeuroImages software (Cox, 1996). The desired cluster size was 15.5 for ROI analysis and 67.3 for the whole-brain gray matter. Using MarsBaR, we extracted the beta estimates from the regions showing a significant interaction effect within the search volume and conducted *post hoc* analyses to confirm the specific pattern of the three-way interaction. Exploratory analyses were performed to identify brain regions showing significant two-way interactions and main effects. In addition, we further explored neural regions associated with the valence level of trait adjectives during each of the observation, control, negative, positive, self and friend condition.

## Results

### Behavioral results

**Fig. 2.**
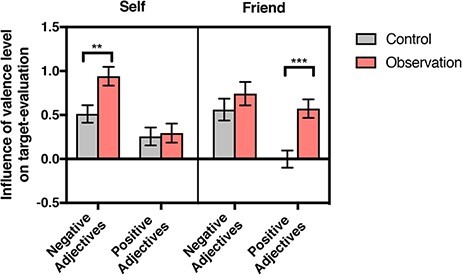
Behavioral results. Bar graphs showing the degrees to which the valence level of trait adjectives affected self- and friend-evaluation. A significant three-way (i.e. Group $ \times $ Target $ \times $ Valence) interaction effect indicates that the observation group (*vs* control group) displayed less negative self-evaluation and more positive friend-evaluation (**P < 0.01, ***P < 0.001).

The behavioral results revealed a significant three-way interaction [*F*(1,41) =* *5.24, *P* = 0.027] (Figure 2). *Post hoc* analysis indicated that OBS participants were more likely than CON participants to disagree with adjectives as the level of negativity increased during self-evaluation [*F*(1,41) = 8.82, *P* = 0.005] and were more likely to agree with adjectives as the level of positivity increased during friend-evaluation [*F*(1,41) = 16.051, *P* < 0.001]. There was a significant main effect of group [*F*(1,41) = 14.66, *P *< 0.001], indicating that OBS participants exhibited a greater tendency to demote a negative target evaluation or promote a positive target evaluation. A significant main effect of valence [*F*(1,41) = 31.02, *P* < 0.001] indicated a greater tendency to demote a negative evaluation than to promote a positive evaluation. The descriptive statistics of the mean ratings for the SN, SP, FN and FP conditions in the OBS and CON groups are presented in Supplementary Table 3. As our main analysis investigated the impact of the valence level on ratings within SN, SP, SN and FP trials, for illustrative purposes, we also presented the mean ratings for the high, middle and low valence levels (Supplementary Figure 2 and Supplementary Table 3).

### Results from independent sample data

**Fig. 3.**
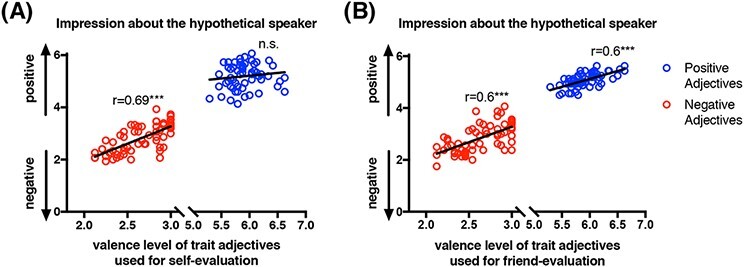
Impression rating data from independent sample data. (A) Impression ratings during self-evaluation: Participants from the independent sample formed a more negative impression of a hypothetical speaker who used more negative trait adjectives for self-evaluation, whereas they did not necessarily form a more positive impression of a hypothetical speaker who used more positive trait adjectives for self-evaluation (***P < 0.001). (B) Impression ratings during friend-evaluation: Participants from the independent sample formed a more negative impression of a hypothetical speaker who used more negative trait-adjectives for friend-evaluation and formed a more positive impression of a hypothetical speaker who used more positive trait-adjectives for friend-evaluation (***P < 0.001).

The analysis of independent sample data was conducted to confirm that the observation effects found in the behavioral results reflect strategic efforts toward impression management. The mean impression of the hypothetical speaker significantly decreased as a function of the valence level of negative adjectives the speaker used for self-evaluation (*r *= 0.69, *P *< 0.0001; Figure 3A), whereas the mean impression was not associated with the valence level of positive adjectives the speaker used for self-evaluation (*r *= 0.13, *P *= 0.32; Figure 3A). The mean impression significantly decreased as a function of the valence level of negative adjectives the speaker used for friend-evaluation (*r *= 0.6, *P *< 0.0001; Figure 3B) and increased as a function of the valence level of positive adjectives the speaker used for friend-evaluation (*r *= 0.64, *P *< 0.0001; Figure 3B). This suggests that decreasing the negative evaluation of both self and a friend, and increasing the positive evaluation of a friend, could serve the goal of impression management.

### fMRI results

**Fig. 4.**
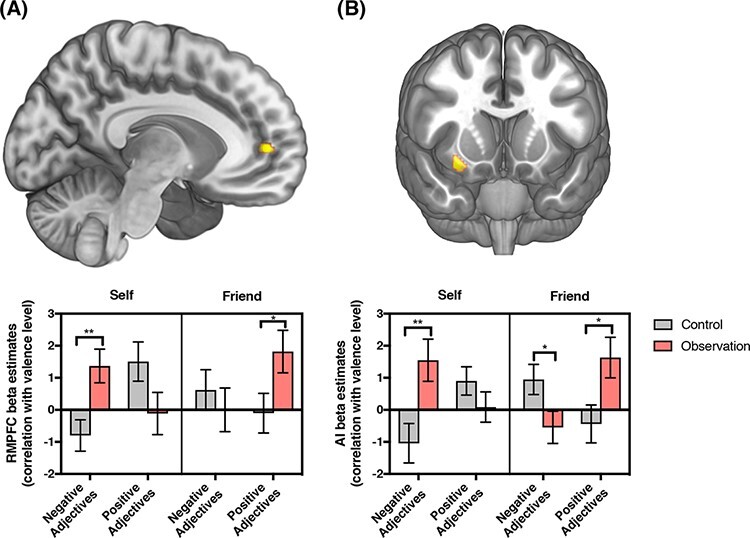
fMRI Results. (A) The rmPFC cluster (peak coordinates: −12, 48, 6; upper panel) showing a significant three-way interaction effect in the voxel-wise three-way ANOVA, and a bar graph (bottom panel) showing condition-specific parameter estimates in the cluster (*P < 0.05, **P < 0.01). (B) The insula cluster (peak coordinates: 30, 14, −14; upper panel) showing a significant three-way interaction effect in the voxel-wise three-way ANOVA, and a bar graph (bottom panel) showing condition-specific parameter estimates in the cluster (*P < 0.05, **P < 0.01).

Analysis using the three-way interaction contrast {i.e. Self [OBS (Neg − Pos) − CON (Neg − Pos)] − Friend [OBS (Neg − Pos) − CON (Neg − Pos)]} showed significant clusters in the rmPFC (peak coordinate = −12, 48, 6; cluster size = 17; small-volume family-wise-error-corrected (SVC-FWE) *P* < 0.05) (Figure 4A) and AI (peak coordinate = 30, 14, −14; cluster size = 26; SVC-FWE *P* < 0.05) (Figure 4B). *Post hoc* analyses revealed that the rmPFC and AI activity in the OBS *vs* the CON group was more strongly correlated with the valence level of negative adjectives during self-evaluation [rmPFC: *F*(1, 41) = 9.089, *P* = 0.004; AI: *F*(1,41) = 8.295, *P* = 0.006] and the valence level of positive adjectives during friend-evaluation [rmPFC: *F*(1, 41) = 4.499, *P* = 0.04; AI: *F*(1, 41) = 5.727, *P* = 0.021], which was consistent with the behavioral results. In addition, the CON group showed greater AI activity than the OBS group when tracking the valence level of negative adjectives during friend-evaluation [*F*(41, 1) = 4.689, *P* = 0.036]. Whole-brain analyses revealed no significant regions. Results with a lenient threshold (i.e. uncorrected *P*-value < 0.001, cluster size $ \ge $10) are reported in Supplementary Table 4.

Exploratory analyses of two-way interactions and main effects showed no significant regions. We found significant rmPFC activity correlating with valence level in observation, self, friend, negative and positive conditions (Supplementary Figure 3).

## Discussion

This study aimed to investigate the behavioral manifestation and neural mechanism of impression management during self- and friend-evaluation under social observation. The behavioral results showed that people evaluated themselves and friends in a socially desirable way under social observation. Specifically, OBS participants demoted negative evaluation of self and promoted positive evaluation of their friends, compared to the CON group. An independent behavioral study confirmed that this behavioral strategy serves the goal of transmitting a favorable self-image to observers by showing that people form a better impression of a speaker who evaluates oneself with less negative adjectives and one’s friend with more positive adjectives. Neuroimaging analysis revealed that the OBS group (*vs* CON group) showed more rmPFC and AI activity when tracking the valence level of negative adjectives during self-evaluation and the valence level of positive adjectives during friend-evaluation. This result suggests that the rmPFC and AI contribute to impression management, playing critical roles in computing the value of preventing an unfavorable impression and promoting a favorable self-image on a trial-by-trial basis.

### Impression management during self- and friend-evaluation

#### Self-evaluation.

Social observation reduced negative self-evaluation but did not affect positive self-evaluation. Consistent with this, the independent behavioral data revealed that people prefer those who evaluate themselves less negatively, but not those who evaluate themselves more positively, as indicated by the nonsignificant linear correlation. Reduced negative self-evaluation during observation is consistent with previous findings that people rarely describe themselves negatively in front of others (Schütz, 1997) and that individuals who describe themselves too negatively are less likable than balanced self-describers (Schlenker and Leary, 1982; Robinson *et al.*, 1995) and those who overstate one’s weakness are viewed as socially unattractive, potentially due to inferred irresponsibility (Lai *et al.*, 2010; Wang and Highhouse, 2016).

The absence of an observation effect in positive self-evaluation and the absence of a modulatory effect in positive self-evaluation on impression formation are inconsistent with previous studies showing that, in public, people reduce positive self-evaluation (Miller and Schlenker, 1985; Baumeister and Ilko, 1995; Exline and Lobel, 1999; Sedikides *et al.*, 2002) and self-enhancing individuals are less liked than modest self-presenters (Bond *et al.*, 1982; Schlenker and Leary, 1982; Robinson *et al.*, 1995). This inconsistency could be attributed to the difference in the context of self-evaluation: while our task included both competence-related and warmth-related words, people evaluated themselves in the competence-related context in the previous studies reporting decreased positive self-evaluation to convey favorable impressions.

#### Friend-evaluation.

Social observation increased positive friend-evaluation, consistent with the result from the independent sample that people prefer those who evaluate their friends more positively. These findings are in line with previous findings that people increase other-enhancing behavior in public (Miller and Schlenker, 1985; Baumeister and Ilko, 1995) and people who show a modesty bias, which often includes other-enhancement (Chen *et al.*, 2009), are well liked by others (Diekmann *et al.*, 2015; Wang and Highhouse, 2016). The present finding that observation did not reduce negative friend-evaluation seems inconsistent with the result from the independent study that people who speak unwell of others were perceived less favorably. A possible explanation for such a discrepancy could be that avoiding a negative friend-evaluation is fully internalized, such that it can be entertained even in private situations (i.e. CON group), or that participants may have chosen a friend who does not possess severe enough negative traits for the trait-evaluation task, which could lead to a ceiling effect in avoiding negative friend-evaluation.

### rmPFC function in impression management

OBS participants, compared to CON participants, showed increased rmPFC activity, tracking the valence level of trait adjectives during negative self-evaluation and positive friend-evaluation. The observed role of rmPFC activity in impression management is consistent with its activity during social observation (Izuma *et al.*, 2010; Somerville *et al.*, 2013; Müller-Pinzler *et al.*, 2015; Van Hoorn *et al.*, 2016; Jung *et al.*, 2018), during the experience of self-conscious emotions (Takahashi *et al.*, 2004; Basile *et al.*, 2011; Wagner *et al.*, 2011; Michl *et al.*, 2012; Gilead *et al.*, 2016) and when engaging in socially desirable behavior (Hare *et al.*, 2010; Rameson *et al.*, 2012; Tusche *et al.*, 2016). Moreover, our results are consistent with recent studies that have demonstrated the role of this region in dynamically monitoring self-value in the eyes of others (Will *et al.*, 2017; Yoon *et al.*, 2018).

Importantly, even in the absence of social observation, rmPFC activity has been strongly implicated in trait judgment (Van Overwalle, 2009; Schurz *et al.*, 2014), especially for the self and close others (Vanderwal *et al.*, 2008; Murray *et al.*, 2012; Araujo *et al.*, 2015). Although several studies have linked rmPFC activity during trait judgment to similarity (Mitchell *et al.*, 2006), closeness (Krienen *et al.*, 2010) and knowledge (Welborn and Lieberman, 2015), the specific role of the rmPFC in trait judgment remains disputed. By showing that social observation facilitates rmPFC function, which is critical for impression management, our study provides a novel view that the increased rmPFC activity during trait-judgment of self and close others reported in previous studies may reflect its allostatic regulatory function largely related to the motivation for socially desirable target evaluation. Participants in a typical trait-judgment task may be concerned with how others (e.g. an MRI operator or an experimenter) would think about their judgments. Alternatively, participants may be concerned about whether their trait judgments are aligned with social expectations, even without being conscious of an observer. Despite the difficulty of measuring private concerns about social desirability, future studies should more systematically elucidate the effect of motivation for social desirability on rmPFC activity during trait-judgment tasks. Specifically, this can be achieved by adding a condition with an instruction that guarantees complete confidentiality of participants’ responses (i.e. a private instruction condition) to examine parametric differences in rmPFC activity across three conditions (i.e. private instruction, no instruction and observation instruction).

The present study also provides novel insights into the role of mPFC in tactical self-enhancement or a strategic enhancement of oneself in consideration of social norms and long-term social interests (see Sedikides and Gregg, 2008; Cai *et al.*, 2011; Sedikides *et al.*, 2015). The function of the medial orbitofrontal cortex (mOFC) has been implicated in self-enhancement, such as evaluating oneself better than average others (Beer and Hughes, 2010; Hughes and Beer, 2013) and in self-serving behavior in the context of accountable *vs* unaccountable conditions (Hughes and Beer, 2012). Extending these studies, the present findings suggest that the rmPFC function could also be associated with a tactical and sophisticated form of self-enhancement modulated by social observation. Future studies may investigate the possibility of functionally dissociable roles of mOFC and rmPFC in different forms of self-enhancement.

### Coactivation of the rmPFC and AI during impression management

In addition to the rmPFC, the AI was also engaged in impression management during self- and friend-evaluation. AI has been theorized as a central hub of emotion processing (Damasio, 1999; Craig and Craig, 2009), playing a key role in integrating bodily signals and linking them to higher-order cognition, which is critical for adjusting behaviors adaptively depending on emotional and social contexts (Ibañez and Manes, 2012). Moreover, structural impairment in these two regions was associated with reduced self-conscious emotions and socially inappropriate behavior (Rosen *et al.*, 2002; Seeley, 2010; Ibañez and Manes, 2012).

It has been theorized that the rmPFC and AI may comprise key neurocircuitry for visceromotor prediction or allostatic regulatory control (Stephan *et al.*, 2016; Kleckner *et al.*, 2017). These two regions are known to communicate directly with the midbrain homeostatic control system in the brainstem (Fischer *et al.*, 2016), possibly via von Economo neurons, which are known to support rapid brain–body communications (Allman *et al.*, 2010, 2011). According to this view, the function of the rmPFC and AI would be to predict potential future homeostatic imbalance and prompt preventive physiological responses or behavioral actions. Impression management in our study may be the behavioral manifestation of allostatic regulatory processes, subserved by the rmPFC and AI to prevent foreseen homeostatic disturbances frequently accompanied by social threat (Dickerson *et al.*, 2009; Slavich *et al.*, 2010). For example, during friend-evaluation with positive adjectives, knowledge leading to rejection of positive traits will clash with expected social rewards that typically follow praise for others. This conflict could be detected by the AI and communicated to the rmPFC and elicited socially desirable friend-enhancement. We believe that allostatic regulatory control parsimoniously explains rmPFC and/or AI activity observed in studies regarding social observation, socially desirable behavior and self-conscious emotions, which all share a common psychological process—concern for potential social evaluation.

### Limitation

The current study has two major limitations: (1) the sample size is smaller than the recent recommendation for task-based fMRI research (Geuter *et al.*, 2018) and (2) the lack of normality of the positive and negative adjectives in terms of the domain (e.g. morality, sociality, competence, etc.) and the distance from the reference point. Future research with a larger sample size and more careful sampling of adjectives is necessary to confirm the roles of mPFC and AI in impression management during negative self-evaluation and positive friend-evaluation. Moreover, it should be emphasized that the current study limited the other as the fifth closest friend. Future studies will explore whether our neural findings can be generalized when participants evaluate more distant or closer others. Further discussion of inconsistencies between behavioral analyses using valence level and impression ratings is provided in Supplementary Text 7.

## Conclusion

Social observation motivates people to demote negative self-evaluation and promote positive friend-evaluation, serving the common goal of communicating positive self-image or impression management, which appears to be served by the rmPFC and AI activity tracking the valence level of trait adjectives. This study provides important insights into our understanding of impression management according to social context, a fundamental skill for building social connections, and a potential core basis of complex arrays of human social behavior.

## Supplementary Material

nsab008_Supp

## Data Availability

Unthresholded maps of Figure 4, Supplementary Figure 3 and Supplementary Figure 4 are available at Neurovault (https://neurovault.org/collections/BJOVOATN/).
