# Supplementary material for: Roles of the MPFC and insula in impression management under social observation
Source: Soc Cogn Affect Neurosci. 2021 Jan 15;16(5):474–83. doi: 10.1093/scan/nsab008 (PMC8095000; doi:10.1093/scan/nsab008)
Supplement: nsab008_Supp [file nsab008_supp.zip › Supplementary Material.docx]

**Supplementary Material**

**Supplementary Text 1. Explanation for determining sample size.**

We performed a power analysis with the G*Power software to ensure that the present study had a sufficient sample size to obtain statistically meaningful results. Assuming a medium effect size of 0.06 and an alpha level of 0.05, to achieve a power of 0.90 for a mixed ANOVA with 2 groups and 4 repeated measurements, the G*Power analysis yielded a required sample size of 30, that is, 15 participants per group. In addition, we referred to two previous fMRI studies on the neural mechanism of heightened sensitivity to social evaluation, which are closely related to the present study (Heitmann *et al.*, 2014; Peterburs *et al.*, 2016). These studies also used a between-subjects design with 16 or 18 participants for each group.

**Supplementary Text 2. The observation effect in the target-evaluation in the fMRI sample as a function of the impression ratings of the adjectives collected from the independent sample.**

We conducted the same subject-level and group-level analyses as those used for our main behavioral analysis, except that we used the impression ratings of the adjectives obtained from the independent sample as the regressors of the subject-level regression model.

<Subject-level Analysis>

$y=\beta_{IM\_SN}x_{IM\_SN}+\beta_{0}$ Equation (1)

$y=\beta_{IM\_SP}x_{IM\_SP}+\beta_{0}$ Equation (2)

$y=\beta_{IM\_FN}x_{IM\_FN}+\beta_{0}$ Equation (3)

$y=\beta_{IM\_FP}x_{IM\_FP}+\beta_{0}$ Equation (4)

For the subject-level analysis, we ran four linear regression analyses (Equations (1) – (4)) to estimate the extent to which the impression ratings of the trait adjectives for SN, SP, FN, and FP conditions (i.e., IM_SN, IM_SP, IM_FN, IM_FP) obtained from the independent sample influenced participants’ target-evaluation decisions. In each model, $y$, $x$, and $\beta_{0}$ denote the participants’ target-evaluation decision, the impression rating of the presented adjective obtained from the independent sample, and the intercept, respectively. The estimate of $\beta$ (i.e., $\hat{\beta}$) indicates the degree to which the participant’s target-evaluation was modulated by the impression rating of the given adjective. Specifically, high $\hat{\beta}$ indicates that a participant’ decision was more likely to be influenced by the degree to which target-evaluation with a given adjective contributes to a favorable or unfavorable impression. Likewise, low $\hat{\beta}$ indicates that participants were less likely to be influenced by the degree to which target-evaluation with a given adjective contributes to a favorable or unfavorable impression.

<Group-level analysis: *Three-way Analysis of Variance (ANOVA****)***>

For the group-level analysis, four parameter estimates ($\hat{\beta}_{IM\_SN}, \hat{\beta}_{IM\_SP}, \hat{\beta}_{IM\_FN}, \hat{\beta}_{IM\_FP}$) from each participant were entered into a mixed ANOVA with group (OBS or CON) as the between-subject factor, and target (self or friend) and valence (negative or positive) as the within-subject factors. Post-hoc analyses were conducted to confirm the observation effects in specific conditions. IBM SPSS 23.0 statistical software was used for this analysis.

<Results>

The results revealed a significant three-way interaction (*F*(1,41) = 6.23, *p* = 0.017) (see **Figure** below). Post-hoc analysis indicated that the observation effect was significant only in the Friend-Positive condition. Specifically, the decisions were more modulated by the impression value of adjectives during the evaluation of the friend with positive adjectives more in the OBS than in the CON group (*F*(1,41) = 10.686, *p* = 0.002). There was a significant main effect of observation (*F*(1,41) = 6.462, *p* = 0.015), an interaction effect of observation * valence (*F*(1,41) = 5.919, *p* = 0.019), and an interaction effect of observation * target (*F*(1,41) = 4.442, *p* = 0.041).

**Supplementary Text 3. The questionnaire that requires the brief profile of the fifth closest friend.**

The friend to be evaluated on the task is your fifth closest friend, you think. The friend could be either a same-sex or opposite-sex friend. Please write the brief profile of your fifth close friend below. The information will be discarded immediately after the experiment.

1. Friend's Name (Initial) / Gender / Age

2. How you and your friend get to know each other?

3. How long have you and your friend got along with each other?

4. How often you and your friend contact each other, regardless of the means of communication (e.g., telephone, messenger, or social networking service)?

5. How many times did you and your friend meet each other during the last one year?

**Supplementary Text 4. fMRI data acquisition procedures.**

Neuroimaging data were acquired using a Siemens MAGNETOM Trio, a 3T Tim system with a 12-channel head matrix coil. We obtained T1-weighted anatomical images (repetition time [TR] = 1900 ms; echo time [TE] = 2.52 ms; flip angle [FA] = 9°; 1 × 1 × 1 mm in-plane resolution; 256 × 256 matrix size). T2*-weighted functional images were obtained using gradient-echo echo-planar pulse sequences (TR = 2000 ms; TE = 30 ms; FA = 90°; field-of-view (FOV) = 220 mm; 78 × 78 matrix; 36 slices; voxel size = 2.8 × 2.8 × 3.0 mm). Experimental stimuli were presented on an MR-compatible LCD monitor mounted on a head coil (refresh rate: 85 Hz; display resolution: 800 × 600 pixels). Participants used a response grip to respond during the task.

**Supplementary Text 5. The boundaries of the ROIs in MNI coordinates.**

The RMPFC mask boundaries were -17 < x < 17, 36 < y < 72, and -10 < z < 20. The AI mask boundaries were -46 < x < -27 (left), 27 < x < 46 (right), -12 < y < 24, and -20 < z < 8.

**Supplementary Text 6. The possible alternative fMRI analysis.**

The alternative way of assessing the neural mechanisms of impression management would be to measure neural activity correlating with participants’ actual decisions (see **Supplementary Table 4** for the result from this analysis). However, this parameter cannot distinguish between the motivation for impression management and actual beliefs about oneself and a friend. Therefore, we believed that the best way to identify the neural correlates of impression management is to measure neural activity encoding valence level, which is shown to have significant effects on managing as well as forming impression at the behavioral level.

**Supplementary Text 7. Limitation regarding the mismatch between behavioral analysis using valence level and impression ratings.**

Additional analyses on the behavioral data using the impression ratings from the independent sample showed that a significant observation effect was found only in the friend-evaluation with positive traits, but not in the self-evaluation with negative traits. Importantly, however, we should be cautious when interpreting the differences from the direct comparison between self- and other-evaluation, given that the impression ratings are not identical between self- and other-evaluation. One possible explanation for this would be that such a difference between the valence rating effect and the impression rating effect reflects the degree of intentionality of or conscious effort for impression management. For example, strategically switching decisions based on valence of words depending on social context (i.e., observation), may require a rather high level of intentionality or conscious effort. On the other hand, the impression rating effects may be the results from rather intuitive and automatic impression management, because a similar level of effect was observed regardless of observational context in SN condition. In addition, at a more lenient threshold (uncorrected *p* < .001), our supplementary parametric modulation analysis (**Supplementary Figure 4**) with the parameter of impression ratings showed that the rmPFC activity tracked the impression ratings more significantly in the OBS vs. CON group only in the FP condition where observation modulated the impression rating effect. This provides further evidence for the role of the RMPFC in impression management under social observation.

**Supplementary Figure 1. Visualizations of Regions of Interest used as masks for the fMRI analysis.** (A) RMPFC mask (B) AI mask.

**
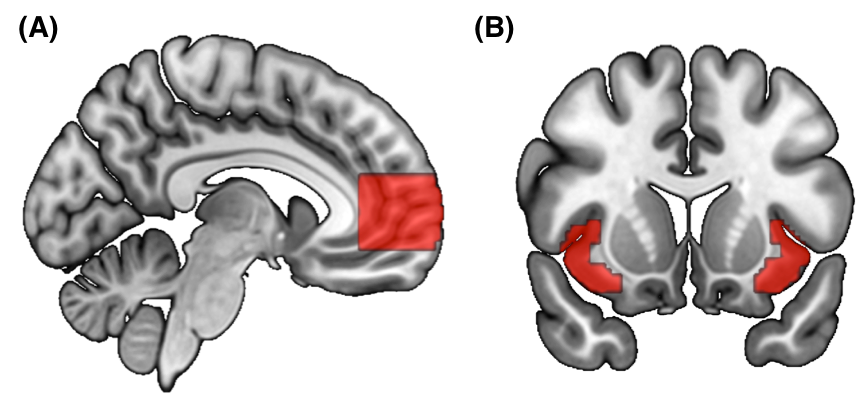
**

**Supplementary Figure 2. Mean ratings for Self-Negative, Self-Positive, Friend-Negative, Friend-Positive condition and three divided valence level of each condition.** Mean and standard deviation of ratings are presented across all trials as well as 10 trials of binned 3 valence level (i.e., high positive/negative, middle positive/negative, and low positive/negative valence level). Note that ratings for Self-Negative and Friend-Negative trials were reverse coded, thereby higher ratings indicate more disagreement to the adjectives.

**Supplementary Figure 3. Significant clusters correlating with valence level of adjectives found in Observation, Self, Friend, Negative, and Positive condition.** (A) MPFC activity (k = 91, [-6, 42, 4], SVC-FWE *p* < .05) showed a significant correlation with valence level in observation group (B) MPFC activity (k = 16, [10, 42, 6], SVC-FWE *p* < .05) showed a significant correlation with valence level during self-evaluation (C) MPFC activity (k = 39, [-4, 40, 10], SVC-FWE *p* < .05) showed a significant correlation with valence level during friend-evaluation (D) MPFC activity (k = 16, [-6, 52, 14], SVC-FWE *p* < .05) showed a significant correlation with negative valence level (E) MPFC activity (k = 80, [-8, 42, 4], SVC-FWE *p* < .05) showed a significant correlation with positive valence level. Note that the valence level for the negative adjectives was reverse coded; therefore, higher valence level for positive and negative adjectives indicate more positive and negative valence level, respectively.

**
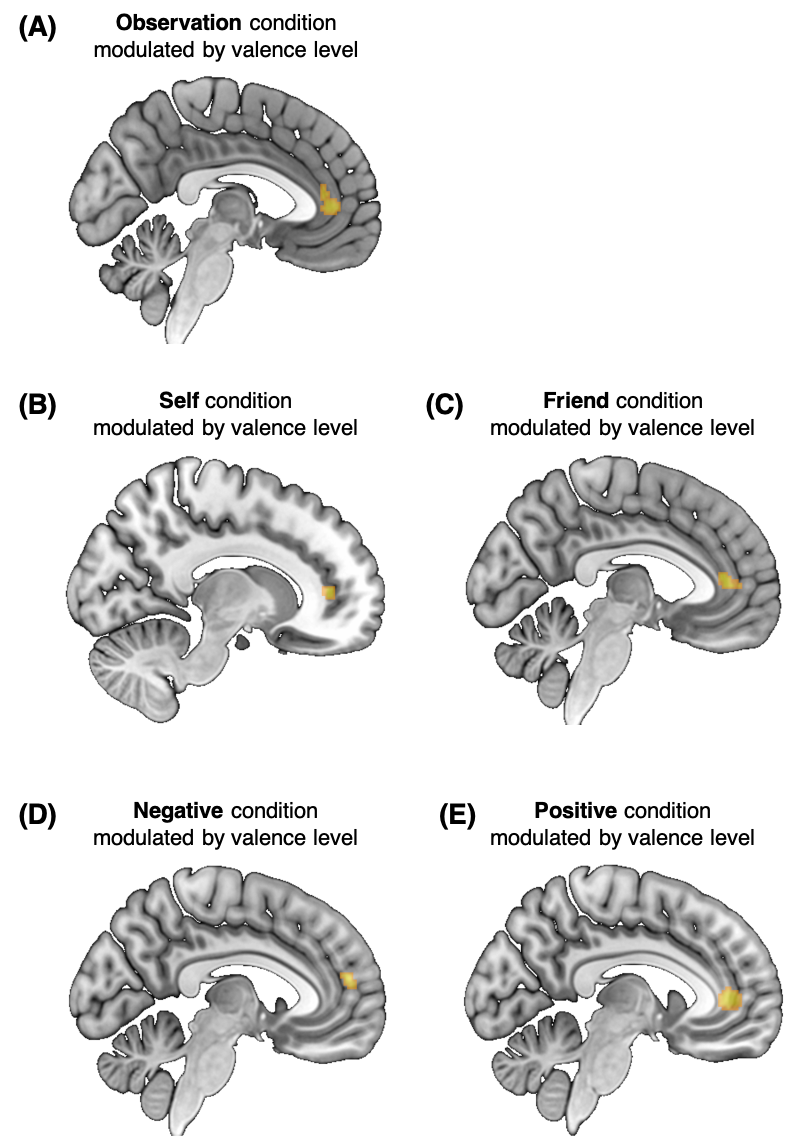
**

**Supplementary Figure 4. The RMPFC activity (k = 6, [-6, 40, 2]) tracking impression ratings during FP condition that showed higher activity in the OBS vs. CON group.**The procedure for building a first-level GLM model was the same as a main parametric modulation analysis, except that the parameter of impression ratings obtained from the independent sample was used instead of the valence level. The search volume was RMPFC+AI, and the statistical threshold was uncorrected *p* < .001.

**
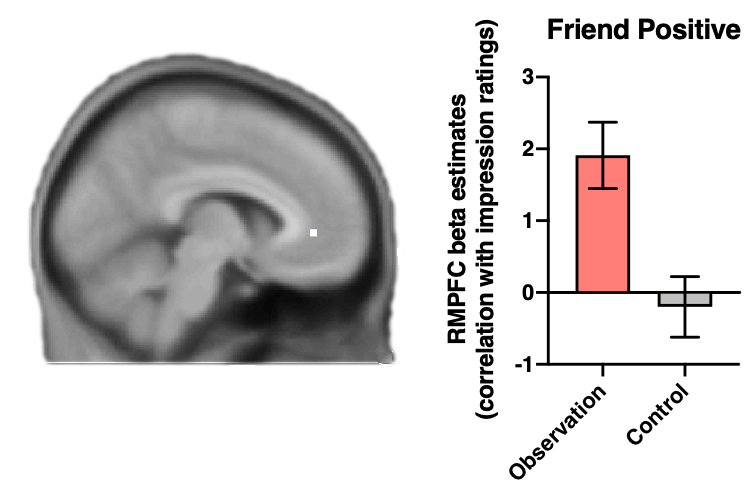
**

**Supplementary Table 1. Descriptive statistics of valence ratings for negative, positive, and moderate (filler) adjectives and example adjectives.**Mean, standard deviation (std), and range are presented across all adjectives in each valence condition as well as binned 3 valence level (i.e., high, middle, and low valence level) of each valence condition. Thirty adjectives of positive and negative condition were binned into 10 adjectives of low, middle, and high valence level. Likewise, 15 adjectives of moderate filler condition were binned into 5 adjectives of low, middle, and high valence level. High valence level indicates more negative or positive traits within negative or positive/moderate traits, respectively. Low valence level indicates less negative or positive traits within negative or positive/moderate traits, respectively. There were two sets of negative, positive, moderate adjectives presented in Self or Friend condition. In one counterbalancing group, set A and set B were presented in Self and Friend condition, respectively. In another counterbalancing group, set A and set B was presented in Friend and Self condition, respectively.

| Valence | Set | Across All trials | | | | Low Valence Level | | | | Middle Valence Level | | | | High Valence Level | | | |
| --- | --- | --- | --- | --- | --- | --- | --- | --- | --- | --- | --- | --- | --- | --- | --- | --- | --- |
|  |  | Mean | std | Min | Max | Mean | std | Min | Max | Mean | std | Min | Max | Mean | std | Min | Max |
| Negative | A | 2.65 | 0.27 | 2.13 | 3 | 2.94 | 0.05 | 2.88 | 3 | 2.68 | 0.11 | 2.54 | 2.83 | 2.34 | 0.12 | 2.13 | 2.5 |
|  |  |  | | | | opportunistic, submissive, immature | | | | cold, stingy, indecisive | | | | intolerant, greedy, arrogant | | | |
|  | B | 2.61 | 0.28 | 2.13 | 3 | 2.92 | 0.08 | 2.83 | 3 | 2.62 | 0.13 | 2.46 | 2.83 | 2.29 | 0.11 | 2.13 | 2.46 |
|  |  |  | | | | unsociable, stubborn, impulsive | | | | jealous, ignorant, boring | | | | unkind, selfish, antisocial | | | |
| Positive | A | 5.92 | 0.34 | 5.46 | 6.63 | 5.6 | 0.08 | 5.46 | 5.71 | 5.85 | 0.07 | 5.71 | 5.92 | 6.32 | 0.25 | 6 | 6.63 |
|  |  |  | | | | popular, cheerful, cooperative | | | | comfortable, bright, optimistic | | | | reliable, competent, considerate | | | |
|  | B | 5.91 | 0.24 | 5.29 | 6.35 | 5.66 | 0.16 | 5.29 | 5.79 | 5.92 | 0.09 | 5.83 | 6.04 | 6.16 | 0.11 | 6.04 | 6.35 |
|  |  |  | | | | calm, skillful, intellectual | | | | mature, lively, excellent | | | | creative, wise, responsible | | | |
| Moderate  (filler trials) | A | 4.43 | 0.37 | 3.75 | 4.83 | 4 | 0.18 | 3.75 | 4.17 | 4.49 | 0.21 | 4.17 | 4.71 | 4.8 | 0.05 | 4.71 | 4.83 |
|  |  |  | | | | perseverant, taciturn, strict | | | | soft, realistic, literary | | | | careful, bold, shrewd | | | |
|  | B | 4.42 | 0.44 | 3.54 | 4.96 | 3.91 | 0.26 | 3.54 | 4.13 | 4.5 | 0.17 | 4.33 | 4.71 | 4.86 | 0.1 | 4.71 | 4.96 |
|  |  |  | | | | shy, compliant, naïve | | | | silent, normal, philosophical | | | | firm, confident, disinterested | | | |

**Supplementary Table 2. Results from exploratory ROI and whole-brain results of the analysis with a parametric modulator of participants’ rating (i.e., the degree to which they (dis)agree with the self- and friend-description with a given adjective) instead of valence level.**Rating for the negative condition was reverse coded, therefore, significant clusters in each condition (i.e., Self-Negative, Self-Positive, Friend-Negative, Friend-Positive) indicate regions associated with the behavior of target-enhancement or protection. Specifically, significant clusters found in the negative condition indicate the regions encoding greater disagreement, while significant clusters found in the positive condition indicate the neural regions encoding greater agreement. Group-level analyses to search brain regions showing significant 3-way interaction (group * target * valence), 2-way interactions (group * valence, group * target, valence * target), and three main effects (group, valence, target) were performed. We also searched for brain regions that correlated with agreement or disagreement during each condition (i.e., observation, control, negative, positive, self, and friend). Note that there was no significant cluster for our main contrast of interest (i.e., 3-way interaction), where we found significant behavioral findings. All the significant clusters were labeled according to the Automatic Anatomical Labeling version 2 (AAL2) and Anatomy using bspmview toolbox. The statistical threshold was determined using 3dClustSim of Analysis of Functional NeuroImages (AFNI) software. The desired cluster size for surviving multiple comparisons at ɑ < 0.05 was 16.4 and 64.8 for ROI and whole-brain gray matter, respectively.

| Region Label | | Extent | t-value | x | y | z |
| --- | --- | --- | --- | --- | --- | --- |
| AAL2 | Anatomy |  |  |  |  |  |
| ***Search Volume: ROI (RMPFC + AI)*** | | | | | | |
| **2 way interaction**  **(observation * target: OBS-Friend, CON-Self > OBS-Self, CON-Friend)** | | | | | | |
| Cingulate_Ant_R | R Superior Medial Gyrus | 20 | 3.6 | 10 | 48 | 10 |
| **target main effect (friend > self)** | | | | | | |
| Cingulate_Ant_R | R ACC | 17 | 3.85 | 4 | 38 | 16 |
| **observation condition** | | | | | | |
| Cingulate_Ant_R | R ACC | 32 | 3.72 | 12 | 46 | 14 |
| Cingulate_Ant_L | Location not in atlas | 19 | 3.6 | -12 | 48 | -2 |
| **friend condition** | | | | | | |
| Cingulate_Ant_R | R ACC | 58 | 4.65 | 6 | 38 | 18 |
| Frontal_Med_Orb_L | L Mid Orbital Gyrus | 98 | 4.42 | -10 | 52 | -2 |
| Frontal_Med_Orb_R | R Mid Orbital Gyrus | 68 | 4.17 | 6 | 52 | -2 |
| ***Search Volume: Whole-brain Gray Matter*** | | | | | | |
| **valence main effect (positive > negative)** | | | | | | |
| Lingual_R | R Linual Gyrus | 527 | 9.68 | 16 | -66 | -4 |
| **valence main effect (negative > positive)** | | | | | | |
| Lingual_L | L Lingual Gyrus | 215 | 7.1 | -14 | -72 | -4 |
| **observation condition** | | | | | | |
| Cuneus_L | L Cuneus | 406 | 5.412 | -4 | -90 | 28 |
| Occipital_Sup_R | R Cuneus | 406 | 5.243 | 16 | -86 | 32 |
| Occipital_Mid_L | L Middle Occipital Gyrus | 100 | 4.262 | -44 | -72 | 10 |
| **negative condition** | | | | | | |
| Lingual_L | L Lingual Gyrus | 90 | 5.01 | -14 | -70 | -4 |
| **positive condition** | | | | | | |
| Lingual_R | R Linual Gyrus | 183 | 6.572 | 14 | -66 | -2 |
| Frontal_Mid_2_L | L Middle Frontal Gyrus | 127 | 4.258 | -36 | 26 | 38 |
| Supp_Motor_Area_L | L Posterior-Medial Frontal | 72 | 4.11 | -2 | 0 | 52 |

**Supplementary Table 3. Mean (dis)agreement ratings for Self-Negative, Self-Positive, Friend-Negative, Friend-Positive condition of Observation and Control group.** Mean and standard deviation of ratings are presented across all trials as well as 10 trials of three binned valence level (i.e., high, middle, and low valence level). High valence level indicates more negative and positive traits for Negative and Positive condition, respectively. Low valence level indicates less negative and positive traits for Negative and Positive condition, respectively. Note that ratings for Self-Negative and Friend-Negative trials were reverse coded to make higher ratings indicate more disagreement to the adjectives.

|  | | Across all trials (30 trials) | Low Valence Level (10 trials) | Middle Valence Level (10 trials) | High Valence Level (10 trials) |
| --- | --- | --- | --- | --- | --- |
|  |  |  |  |  |  |
| Self-Negative | Observation | 2.94 (0.33) | 2.62 (0.44) | 2.97 (0.37) | 3.22 (0.29) |
|  | Control | 2.85 (0.39) | 2.75 (0.46) | 2.77 (0.39) | 3.03 (0.44) |
| Self-Positive | Observation | 2.88 (0.38) | 2.77 (0.38) | 2.94 (0.45) | 2.94 (0.45) |
|  | Control | 2.78 (0.37) | 2.7 (0.36) | 2.83 (0.45) | 2.82 (0.46) |
| Friend-Negative | Observation | 3.29 (0.35) | 3.1 (0.39) | 3.22 (0.39) | 3.56 (0.41) |
|  | Control | 3.2 (0.28) | 3.05 (0.35) | 3.17 (0.31) | 3.37 (0.39) |
| Friend-Positive | Observation | 2.9 (0.43) | 2.66 (0.48) | 3 (0.47) | 3.03 (0.54) |
|  | Control | 2.87 (0.34) | 2.85 (0.42) | 2.91 (0.45) | 2.84 (0.34) |

**Supplementary Table 4.** **Clusters from** **Whole brain analysis with the lenient threshold (i.e., uncorrected p value < 0.001, cluster size ≥ 10).** Regions were labelled according to the Automatic Anatomical Labeling version 2 (AAL2) and Anatomy using bspmview toolbox.

| Region Label | | Extent | t-value | x | y | z |
| --- | --- | --- | --- | --- | --- | --- |
| AAL2 | Anatomy |  |  |  |  |  |
| Insula_R | R Insula Lobe | 28 | 4.67 | 30 | 14 | -14 |
| Location not in atlas | R Rolandic Operculum | 21 | 4.32 | 44 | -16 | 24 |
| Temporal_Sup_L | L Middle Temporal Gyrus | 18 | 4.2 | -54 | -12 | -6 |
| Vermis_4_5 | Cerebellar Vermis (4/5) | 26 | 4.2 | 2 | -54 | 6 |
| Cingulate_Post_R | Location not in atlas | 10 | 3.64 | 10 | -38 | 8 |
| Frontal_Sup_Medial_L | L Superior Medial Gyrus | 17 | 3.56 | -12 | 48 | 6 |

**Supplementary References**

Heitmann, C. Y., Peterburs, J., Mothes‐Lasch, M., Hallfarth, M. C., Böhme, S., Miltner, W. H. & Straube, T. (2014) 'Neural correlates of anticipation and processing of performance feedback in social anxiety', *Human Brain Mapping*, **35**(12), pp. 6023-6031.

Peterburs, J., Sandrock, C., Miltner, W. H. & Straube, T. (2016) 'Look who's judging-Feedback source modulates brain activation to performance feedback in social anxiety', *NeuroImage*, **133**, pp. 430-437.
